# Muscle Strength Identification Based on Isokinetic Testing and Spine Musculoskeletal Modeling

**DOI:** 10.34133/cbsystems.0113

**Published:** 2024-05-24

**Authors:** Zuming Xiao, Chang Li, Xin Wang, Jianqiao Guo, Qiang Tian

**Affiliations:** ^1^ MOE Key Laboratory of Dynamics and Control of Flight Vehicle, School of Aerospace Engineering, Beijing Institute of Technology, Beijing, China.; ^2^ Professional and Technical Innovation Center for Exercise Diagnosis and Evaluation, Shenyang Sport University, Shenyang, China.

## Abstract

Subject-specific spinal musculoskeletal modeling can help understand the spinal loading mechanism during human locomotion. However, existing literature lacks methods to identify the maximum isometric strength of individual spinal muscles. In this study, a muscle strength identification method combining isokinetic testing and musculoskeletal simulations was proposed, and the influence of muscle synergy and intra-abdominal pressure (IAP) on identified spinal muscle strength was further discussed. A multibody dynamic model of the spinal musculoskeletal system was established and controlled by a feedback controller. Muscle strength parameters were adjusted based on the measured isokinetic moments, and muscle synergy vectors and the IAP piston model were further introduced. The results of five healthy subjects showed that the proposed method successfully identified the subject-specific spinal flexor/extensor strength. Considering the synergistic activations of antagonist muscles improved the correlation between the simulated and measured spinal moments, and the introduction of IAP slightly increased the identified spinal extensor strength. The established method is beneficial for understanding spinal loading distributions for athletes and patients with sarcopenia.

## Introduction

Spinal musculoskeletal modeling provided a quantitative tool for predicting spinal vertebral motions and muscle loadings during three-dimensional spinal movements [1]. To date, researchers have developed numerous spinal musculoskeletal models, widely used in ergonomics [2–4], sports training [5–6], rehabilitation [7–9], etc. The accuracy of musculoskeletal simulations depended largely on their biomechanical parameters [10]. Among them, the muscle strength parameter was directly related to the predicted activation patterns [10–11] and crucial for musculoskeletal simulation accuracy [12–13].

Previous studies have focused on the parameter identification methods of musculoskeletal models. Among them, the most popular way was to adjust the segment length, mass, and moment of inertia based on the subject-specific height and weight [14–17]. However, the above studies ignored the difference of muscular biomechanical behavior. With the help of medical imaging techniques, researchers have proposed identification methods of muscle path [18,19], optimal length, physiological cross-sectional area (PCSA) [17,20], or even joint loading [21], but these methods still could not determine subject-specific muscle strength. Moreover, to determine subject-specific muscle strength, researchers often assumed that its value was linearly related to the corresponding PCSA [15,17,20,22], whereas the strength scaling coefficients could not be identified via imaging.

Muscle strength identification generally requires quantitative experiments such as isokinetic testing. In this condition, researchers often assumed that the joint moment was generated by a single muscle group, neglecting the contribution of its antagonist muscles. However, according to Steele et al. [23] and Torres-Oviedo and Ting [24], muscle strength identification should consider the subject-specific synergistic activations, which could explain the control mechanism of the central nervous system on muscle recruitment [25]. Muscle synergy helped maintain human balance and postural stability [26]. In addition, control strategies considering muscle synergy could also reduce the sensitivity to the uncertainty of muscle parameters [27]. According to Sheng et al. [28], the introduction of synergistic patterns into the musculoskeletal model could improve the accuracy of the predicted spinal movement, while its influence on muscle strength identification has not been discussed.

Another mechanism that possibly generates spinal extension moment was the intra-abdominal pressure (IAP) [29]. When the breathing muscles like diaphragm or transversus abdominis exert spinal muscle forces, they can squeeze the organs, tissues, and gas inside the abdominal cavity, increasing pressure normal to the diaphragm and abdominal muscles. The IAP depends on the body posture, lifted load, stability of movement, and training level [30]. Studies have shown that increasing IAP could reduce muscle force and intervertebral disc (IVD) loading [20,31–32], and different breathing strategy could affect load lifting performances [33]. For example, IAP substantially reduced the compressive force of the IVD when the trunk was tilted forward [34]. IAP could also improve the accuracy of predicted joint reaction forces [35]. However, it was still unclear whether introducing the effect of IAP will influence the accuracy of muscle strength identification.

This study proposed a numerical method to identify subject-specific strength parameters of spinal flexor and extensor muscles based on isokinetic measurements. It also investigated the effects of physiological co-contraction characteristics, including muscle synergy and IAP, on muscle strength identification results. The “Materials and Methods” section established the muscle strength identification method based on isokinetic loading and spinal musculoskeletal modeling. This section also introduced the effect of muscle synergy patterns and IAP into the musculoskeletal model. As a validation, the “Results” section compared identified muscle strength of the spinal flexors and extensors with measured values. It also analyzed the effects of muscle synergy and IAP on the strength calibration results. The “Discussion” section discussed the main findings of this work and finally gave conclusions.

## Materials and Methods

### Experimental design

Five healthy male volunteers (22.9 ± 2.3 years old, body mass index = 22.2 ± 2.3 kg/m^2^) were recruited to trunk isokinetic testing. All subjects had no history of musculoskeletal diseases. Subjects were instructed to avoid vigorous exercise, alcohol consumption, or the intake of medications that could affect neuromuscular function 24 h before the test, and an informed consent form was signed by each subject.

**Fig. 1.**
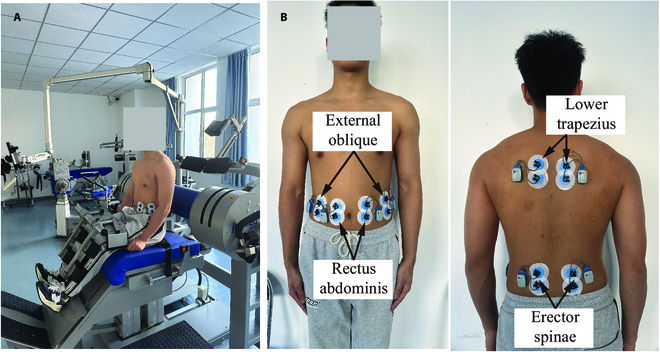
Simultaneous measurement of isokinetic muscle strength and spinal muscle EMG. (A) Isokinetic testing for a typical subject. (B) EMG testing protocol of spinal muscles.

Spinal muscle strength during spinal flexion/flexion was measured by an isokinetic dynamometer (IsoMed2000, D&R Ferstl, Hemnau, Germany; Fig. 1A). A lever with one degree of freedom (DOF) fixed the torso at the shoulder joint. The subject’s lower limb was fixed at femoral and tibial positions using straps. By this means, the subject could only rotate the trunk in the sagittal plane. Before the test, the gravity effect of the trunk at different spinal flexion angles was first recorded and subtracted from isokinetic moment. Then, the subjects were asked to perform five concentric flexion/extension cycles using maximum strength at the angular velocity of 30^°^/s. The spinal movement range was from 30^°^ flexion to 30^°^ extension. The measurement procedure was repeated three times with an interval of 2-min rest to avoid muscle fatigue. The time-dependent spinal moment and corresponded flexion angle were recorded. The obtained moment–time curve was low-pass-filtered through a fourth-order Butterworth filter with a cutoff frequency of 6 Hz [36].

### Synergistic pattern extraction

During the isokinetic testing, surface electromyography (sEMG) signals of the spinal muscles, including the rectus abdominis (RA), external oblique (EO), erector spinae (ES), and lower trapezius on both sides, were simultaneously measured based on a 16-channel recorder (Noraxon, Phoenix, AZ, USA) at the sampling frequency of 2,000 Hz (see Fig. 1B). Here, the number of muscles tested was carefully chosen to reduce interference from neighboring muscle signals. Before applying the electrode pads to the subjects, the skin was cleaned to remove oil, dirt, and hair from the surface to ensure proper adhesion and conductivity. A pair of electrode pads was placed parallel to the tested muscle fiber direction at the highest point of bulging during concentric contraction. The electrode placement was guaranteed to yield optimal sEMG signal using preliminary testing. Moreover, to minimize possible sEMG crosstalk, the absence of electromagnetic signal interference was verified around the testing area, and single-channel and multi-channel sEMG signals were conducted and compared before the isokinetic measurement.

The recorded sEMG signals were filtered using a sixth-order band-pass filter with 10- to 250-Hz cutoff frequencies and full-wave rectified. Then, a fourth-order Butterworth low-pass filter with a cutoff frequency of 6 Hz was applied. Afterward, the signal was normalized by the maximal volumetric contraction (MVC), determined by the maximal sEMG value during spinal flexion/extension isokinetic testing, to obtain the dimensionless neural stimulus *u*. The corresponding muscle activation *a* was calculated using a simplified activation dynamic equation [37–38]:

$$\frac{\mathrm{da}}{\mathrm{dt}}=\frac{u-a}{\tau_{a}\left(a,\;u\right)}$$ (1)

$$\tau_{a}\left(a,\;u\right)=\left\{\begin{array}{cc} \tau_{\mathrm{act}}\left(0.5+1.5a\right) & u>a\\ \tau_{\text{deact}}/\left(0.5+1.5a\right) & u\leq a \end{array}\right.$$ (2)

where *τ_a_*(*a*, *u*) is the characteristic time function, and *τ*_act_, *τ*_deact_ are the activation and deactivation time constants, respectively.

Muscle synergies represented the inter-muscle dependencies generated by the nervous system to perform biomechanical tasks [25]. The non-negative matrix decomposition (NMF) can be used to obtain the subject-specific muscle synergy [39]. By this means, the multi-channel sEMG signal **M**_*X* × *T*_ can be decomposed to synergistic matrix **W**_*X* × *S*_, i.e., the coordinated recruitment of a group of muscles, and the activation signal **H**_*S* × *T*_:

$$M_{X\times T}=W_{X\times S}\times H_{S\times T}+E$$ (3)

Here, *X* is the number of EMG channels, *T* is the sampling point number, *S* is the number of muscle synergies, and **E** is the residual signal. The number of muscle synergies *S* was selected when the total percentage variability accounted for (VAF) exceeded 80% for the first time [40].

### Spinal musculoskeletal model

**Fig. 2.**
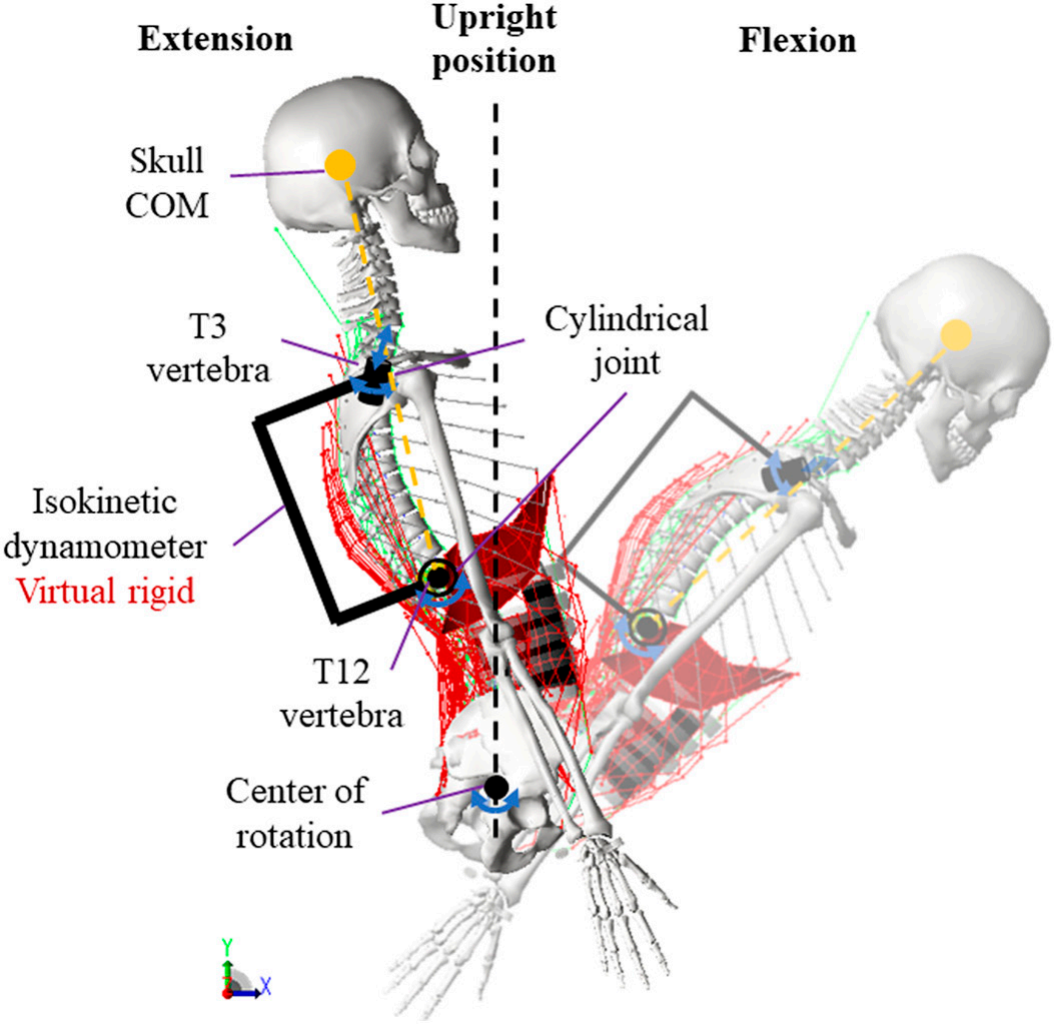
Spinal musculoskeletal model constrained by the isokinetic dynamometer. The spinal flexion is defined based on the sagittal inclination of the line connecting the skull’s center of mass (COM) and that of T12.

The spinal musculoskeletal model consisted of 51 rigid-bone segments (Fig. 2), including the pelvis, sacrum, spinal vertebrate, arms, and skull. Anthropometric data were obtained from a 25-year-old male with 78 kg of mass and 175 cm of height [41]. The skeletal geometry was further assumed to be symmetrical about the sagittal plane. The cervical spine and head masses were taken from Zanoni et al. [42]. The isokinetic dynamometer was simplified as a massless rigid body. We assumed that the T12 vertebra of the musculoskeletal model was connected to the dynamometer through a cylindrical joint, which only allowed sagittal flexion/extension between T12 and the virtual dynamometer (see Fig. 2).

The musculoskeletal model also included the spinal muscle groups to provide postural stability and drive the spinal movements. Each muscle group was divided into multiple fascicles according to its insertion positions:

1. Flexor: RA (2 fascicles), psoas major (PM, 22 fascicles)
2. Extensor: ES (76 fascicles), multifidus (MF; 50 fascicles)
3. Lateral bending: quadratus lumborum (QL; 36 fascicles)
4. Rotation: EO (16 fascicles), internal oblique (IO, 12 fascicles)

**Fig. 3.**
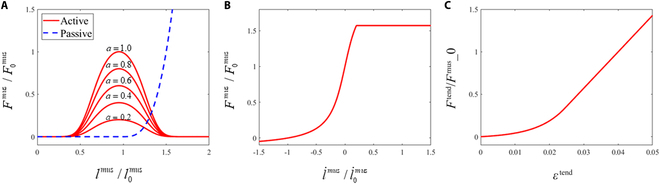
Hill-type muscle model. (A) Muscle force–length curve. (B) Muscle force–velocity curve. (C) Tendon force–strain curve.

Muscle fascicles were discretized by the flexible muscle element based on arbitrary Lagrangian–Eulerian (ALE) description [43–44], which can simultaneously consider the distributed muscular mass, active muscle force, and dynamic muscle wrapping. Here, the sliding joint constraint was utilized to determine the muscle wrapping path [45], ensuring the continuity of muscle material at its via points. A typical Hill-type model (Fig. 3) was introduced to describe the muscle contraction behavior [46]. The muscle model was divided into three force elements: the contractile element (CE) determining the muscular active force, the passive element (PE) describing its nonlinear elastic behavior, and the serial elastic element (SE) corresponding to the tendon stiffness. We also introduced the effect of pennation angle *α*, i.e., the angle between the muscle fiber and its series-connected tendon, in this model [47]. For the pennate muscle, the active contraction force $F_{\mathrm{CE}}^{\mathrm{mus}}$, passive muscle force $F_{\mathrm{PE}}^{\mathrm{mus}}$, and tendon force *F*^tend^ satisfy the following equilibrium equation:

$$\left(F_{\text{CE}}^{\text{mus}}+F_{\text{PE}}^{\text{mus}}\right)\cos\alpha =F^{\text{tend}}$$ (4)

The active contraction force $F_{\text{CE}}^{\text{mus}}$ is determined by the fiber length *l*^mus^, fiber contraction velocity ${\overset{\cdot }{l}}^{\text{mus}}$, and muscle activation *a*:

$$F_{\text{CE}}^{\text{mus}}=a\left(t\right)F_{0}^{\text{mus}}f_{l}\left(l^{\text{mus}},\;l_{0}^{\text{mus}}\right)f_{\dot{l}}\left({\dot{l}}^{\text{mus}},{\dot{\;l}}_{0}^{\text{mus}}\right)$$ (5)

Here, $F_{0}^{\text{mus}}$ is the maximum isometric force, *f_l_* is the correlation between muscle force and fiber length, and $f_{\overset{\cdot }{l}}$ is the correlation between muscle force and fiber contraction velocity [47–49]:

$$\begin{array}{c} f_{l}\left(l^{\text{mus}},l_{0}^{\text{mus}}\right)=\\ \text{exp}\left\{-{\left[\frac{9}{4}\left(\frac{l^{\text{mus}}}{l_{0}^{\text{mus}}}-\frac{19}{20}\right)\right]}^{4}-{\left[\frac{9}{4}\left(\frac{l^{\mathrm{mus}}}{l_{0}^{\text{mus}}}-\frac{19}{20}\right)\right]}^{2}\right\} \end{array}$$ (6)

$$\begin{array}{c} f_{\overset{\cdot }{l}}\left({\overset{\cdot }{l}}^{\mathrm{mus}},{\overset{\cdot }{l}}_{0}^{\text{mus}}\right)=\\ \left\{\begin{array}{cc} 0 & -{\overset{\cdot }{l}}_{0}^{\text{mus}}>{\overset{\cdot }{l}}^{\text{mus}}\\ -\frac{1}{\text{arctan}\left(5\right)}\text{arctan}\left(-5\frac{{\overset{\cdot }{l}}^{\text{mus}}}{{\overset{\cdot }{l}}_{0}^{\text{mus}}}\right)+1 & 0.2{\overset{\cdot }{l}}_{0}^{\text{mus}}\geq {\overset{\cdot }{l}}^{\text{mus}}\geq -{\overset{\cdot }{l}}_{0}^{\text{mus}}\\ \frac{\pi }{4\text{arctan}\left(5\right)}+1 & {\overset{\cdot }{l}}^{\text{mus}}>0.2{\overset{\cdot }{l}}_{0}^{\text{mus}} \end{array}\right. \end{array}$$ (7)

The PE component only produces $F_{\text{PE}}^{\text{mus}}$ when it passively elongates:

$$F_{\text{PE}}^{\text{mus}}=\left\{\begin{array}{cc} 0 & l_{0}^{\text{mus}}>l^{\text{mus}}\\ 8F_{0}^{\text{mus}}{\left(\frac{l^{\text{mus}}}{l_{0}^{\text{mus}}}-1\right)}^{3} & 1.63l_{0}^{\text{mus}}\geq l^{\text{mus}}\geq l_{0}^{\text{mus}}\\ 2F_{0}^{\text{mus}} & l^{\text{mus}}>1.63l_{0}^{\text{mus}} \end{array}\right.$$ (8)

The SE force *F*^tend^ can be described by Thelen [37]:

$$F^{\text{tend}}=\left\{\begin{array}{cc} F_{0}^{\text{mus}}\frac{f_{\text{toe}}^{\text{tend}}}{e^{k_{\text{toe}}}-1}\left(e^{k_{\text{toe}}\varepsilon^{\text{tend}}/\varepsilon_{\text{toe}}^{\text{tend}}}-1\right) & \varepsilon^{\text{tend}}\leq \varepsilon_{\text{toe}}^{\text{tend}}\\ F_{0}^{\text{mus}}\left[k_{\text{lin}}\left(\varepsilon^{\text{tend}}-\varepsilon_{\text{toe}}^{\text{tend}}\right)+f_{\text{toe}}^{\text{tend}}\right] & \varepsilon^{\text{tend}}>\varepsilon_{\text{toe}}^{\text{tend}} \end{array}\right.$$ (9)

where *ε*_toe_ = 0.609*ε*_0_ is the tendon strain above which the tendon exhibits linear force responses, and $f_{\text{toe}}^{\text{tend}}=0.33$ denotes the corresponding normalized tendon force [50]. *k*_toe_ = 3 is an exponential shape factor, and *k*_lin_ = 42.8 is the dimensionless stiffness [37].

### Muscle strength identification

**Fig. 4.**
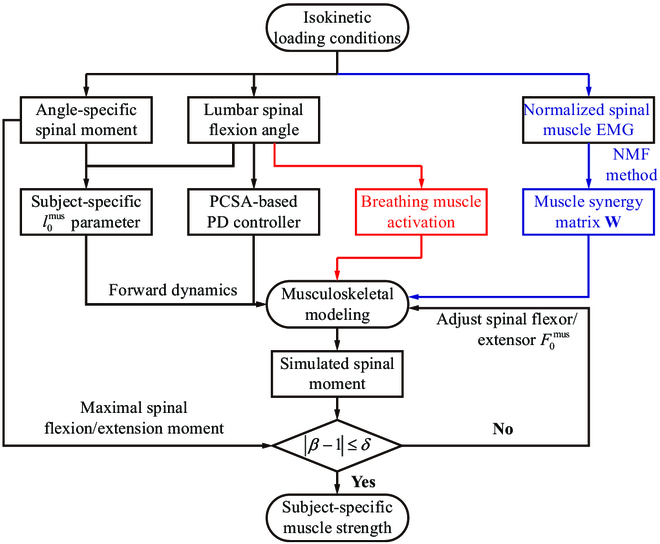
Spinal muscle strength identification based on isokinetic testing and forward dynamic simulations.

To identify the subject-specific spinal muscle strength, the $F_{0}^{\text{mus}}$ of each muscle fascicle within the spinal extensors and flexors was first assumed to be changed synchronously. Their initial values were taken from a generic model [41] based on a linear scaling of PCSA. The forward dynamic simulations were then performed to identify each muscle strength (see Fig. 4). Before the start of isokinetic loading, a 0.5 s initialization was performed to obtain the initial spinal posture. Here, the dynamic relaxation method was used to realize the static equilibrium under gravity loading [51]. Then, the musculoskeletal model was driven by spinal muscles to minimize the error between the simulated and the measured trunk angle ${\tilde{e}}_{g}=e_{g}\left(t-\tau \right)$, where *τ* = 0.5 s denoted the feedforward time corresponding to the neural prediction of future motion [52]. A proportional-derivative (PD) controller was used to calculate the activation *a*_g_ of spinal muscles:

$$a_{g}\left(t\right)=a_{g}\left(t-\Delta t\right)+\frac{{\text{PCSA}}_{m}}{\sum_{i=1}^{n_{g}}P{\mathrm{CSA}}_{i}}\left(k_{g}^{p}{\tilde{e}}_{\theta }+k_{g}^{d}\frac{d{\tilde{e}}_{\theta }}{dt}\right)$$ (10)

where $k_{g}^{p}=0.05$ and $k_{g}^{d}=5.0$ are the proportional and differential gain parameters, respectively, and the sampling rate Δ*t* was taken as 0.01 s. *PCSA_m_* represented the PCSA of muscle *m* in a certain muscle group with *n_g_* fascicles. The obtained activation level *a*_g_ should be normalized between 0.02 and 1.

During forward dynamic simulations, the time-dependent rotation angle of the virtual dynamometer was constrained by the measured values, and the corresponding constraint moment *M*^act^was calculated based on forward dynamic simulations driven by the PD controller. The passive moment *M*^deact^ was generated by the passive extension of spinal muscles, and the difference between *M*^act^ and *M*^deact^ was regarded as the simulated isokinetic moment *M*_isom_. The ratios between the measured and simulated spinal flexion/extension moments $\beta_{\text{mom}}^{\text{ext}}$, $\beta_{\text{mom}}^{\text{flex}}$ were introduced to adjust the muscle strength:

$$\beta_{\text{mom}}^{\text{ext}}=\frac{\text{max}\left(M_{\text{isom}}^{\text{exp}}\right)}{\text{max}\left(M_{\text{isom}}^{\text{simu}}\right)},\;\beta_{\text{mom}}^{\text{flex}}=\frac{\text{min}\left(M_{\text{isom}}^{\text{exp}}\right)}{\text{min}\left(M_{\text{isom}}^{\text{simu}}\right)}$$ (11)

1. If $\beta_{\text{mom}}^{\text{ext}}$, $\beta_{\text{mom}}^{\text{flex}}$ are between 1 − *δ* and 1 + *δ* (*δ* is the threshold value, and a plausible value could be 0.2), the extensor and flexor muscle strength values were regarded consistent with the real-world value and stop calibration.
2. If $\beta_{\text{mom}}^{\text{ext}}$ is lower than 1 − *δ* or higher than 1 + *δ*, the $F_{0}^{m}$ of each fascicle within the spinal extensors should be adjusted to $1/\beta_{\text{mom}}^{\text{ext}}$ of its original value.
3. If $\beta_{\text{mom}}^{\text{flex}}$ is lower than 1 − *δ* or higher than 1 + *δ*, the $F_{0}^{m}$ of each fascicle within the spinal flexors should be adjusted to $1/\beta_{\text{mom}}^{\text{flex}}$ of its original value.

Moreover, to avoid the influence of muscle geometries on strength identification, the optimal fiber lengths of the RA and ES were manually personalized. We assumed that the fiber lengths of these muscles reached their optimal values when they generated maximal $M_{\text{isom}}^{\text{simu}}$. The obtained values at each loading cycle were different, and their average was taken as $l_{0}^{\text{mus}}$.

### Introduction of muscle synergy

In this section, the influence of synergy on neuromuscular control on spinal muscle strength identification was considered. The first two-order muscle activation patterns extracted by NMF, including an extensor-dominated synergistic vector and a flexor-dominated one, were taken from the normalized EMG data and added to the activation controller:

$$a_{m}\left(t\right)=\left\{\begin{array}{cc} \frac{W_{\left(\text{Ext},\;\text{Flx}\right)}}{W_{\left(\text{Flx},\;\text{Flx}\right)}}a_{\text{Ext}}\left(t\right) & m\in \text{Ext},t\in \text{Flx},\\ \frac{W_{\left(\text{Flx},\;\text{Ext}\right)}}{W_{\left(\text{Ext},\;\text{Ext}\right)}}a_{\text{Flx}}\left(t\right) & m\in \text{Flx},t\in \text{Ext},\\ a_{g}\left(t\right)\; & \text{else}. \end{array}\right.$$ (12)

where *t* ∈ Flx represents the flexion phase (Fig. 2), *m* ∈ Ext represents the fascicles within the spinal extensors, *W*_(Ext, Flx)_ represents the proportion of spinal flexors in extensor-dominated synergistic vector, and *a*_Ext_(*t*) represents the predicted spinal extensor activations during spinal extension. By this means, the flexion muscles were activated synergistically, reflecting the antagonist co-contraction during isokinetic movement.

### Introduction of IAP loading

**Fig. 5.**
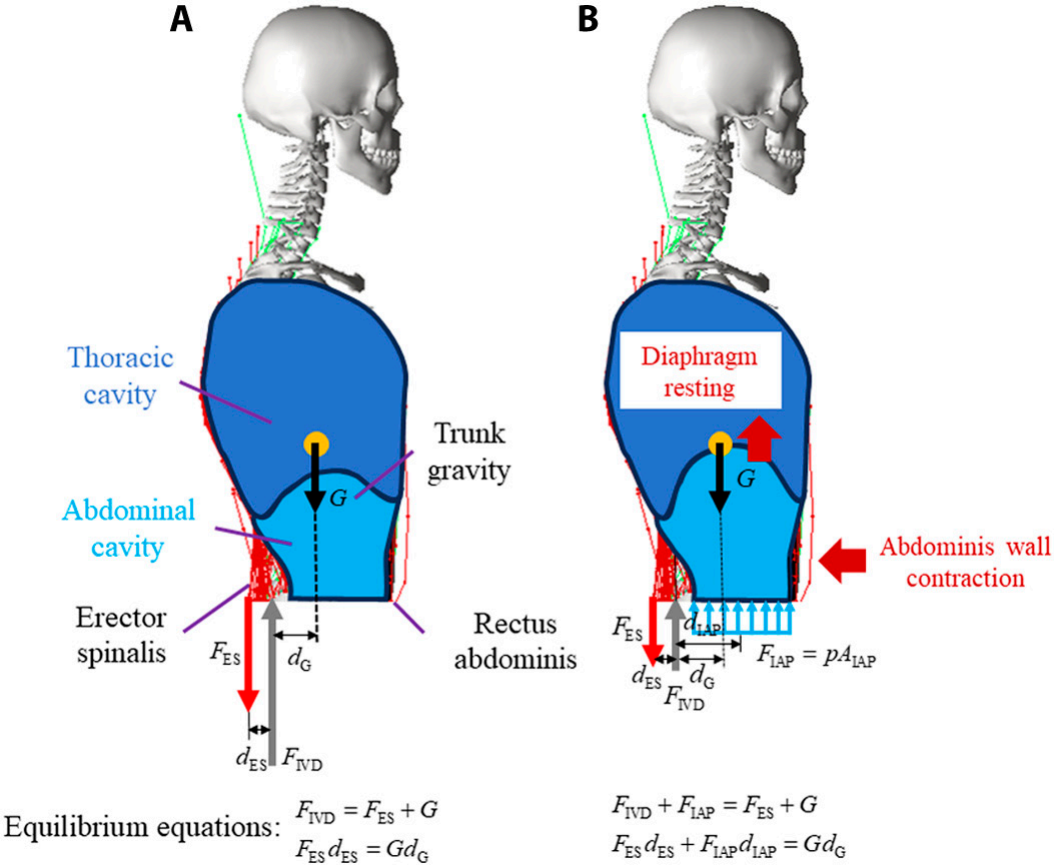
Free body diagrams of the spinal unloading mechanism (A) without IAP and (B) with IAP (*d*_ES_ = the moment arm of erector spinalis; *d*_G_ = the gravity moment arm).

This study further discussed the effect of IAP on muscle strength identification. The breathing muscles, especially for the diaphragm and the transversus abdominis, were involved in abdominal breathing. The influence of pelvic floor muscles on IAP was complex and ignored for simplicity. Following the same procedure in [31], the free body diagram analysis was performed for the human body upper than L3 vertebra to understand the effect of IAP on spinal loading (Fig. 5). Without considering IAP, the ES force was higher than the trunk gravity because the ES moment arm *d*_ES_ is much smaller than that of gravity *d*_G_. After adding IAP, the resultant force erected on the diaphragm formed a couple with the ES. Meanwhile, the IAP increase did not change the distances between the gravity loading and the IVD force. As a result, the ES force should be decreased due to its moment arm increase.

Therefore, to increase the obtained spinal extension moment, it was assumed that the subject inhaled during *t* ∈ Flx and exhaled during *t* ∈ Ext.

1. During the inhalation (flexion) phase, the neural excitation *u* of the diaphragm was assumed linearly increased to tonic activation in the first 0.5 s and maintained tonic activation until 0.5 s before exhalation.

2. During the exhalation (extension) phase, the activation patterns of the transversus abdominis were consistent with those of the diaphragm during the inhalation phase.

### Simulation and statistical analysis

The musculoskeletal dynamic modeling was implemented based on our in-house C/C++ code. All the numerical simulations were performed using an Intel Xeon dual-core processor with four threads. As a preliminary verification, the identified muscle strength in the first trial was used as the corresponding muscle parameters in the subsequent two trials of the same subject. The identified isokinetic strength error was determined as the difference of the average of the first three maximum moment values between the identification results and experimental measurements. Pearson correlation coefficients (*R* values) were calculated to explore the correlation between muscle strength predictions and experimental values of each subject. The root mean square error (RMSE) was used to illustrate their deviations, denoted as mean ± standard deviations (*μ* ± *σ*).

## Results

### Subject-specific isokinetic strength

The predicted spinal flexion angle was almost identical to the measured values (RMSE = 1.43 ± 0.59^∘^), and the error was because the PD controller cannot totally eliminate the offset of the spinal moment prediction results. The Pearson’s *R* between the measured and simulated moment results was 0.79 ± 0.04. Moreover, the obtained maximum muscle strength did not change significantly whether $l_{0}^{\mathrm{mus}}$ of the RA and ES was individually adjusted.

The measured maximum extension moment was significantly greater than the flexion one, because the spinal extensors need to resist gravity of the upper body. Although the measured maximum muscle strength was different in various trials due to fatigue and neuroplasticity, the isokinetic strength identification method successfully reduced the error between the measured and simulated maximum moment to less than 20% for each trial (Table 1).

**Table 1.** Comparison of the subject-specific muscle strength identification results

Subject	center		Correlation coefficient	Erector spinae		Rectus abdominis	
				Maximum moment (Nm)	Error (%)	Maximum moment (Nm)	Error (%)
1	Identification	Experiment	0.772	194.30	14.79	170.56	6.67
		Simulation		223.03		181.93	
	Verification 1	Experiment	0.740	181.57	0.97	155.94	14.57
		Simulation		183.32		178.65	
	Verification 2	Experiment	0.773	196.97	7.25	157.02	14.97
		Simulation		182.69		180.54	
2	Identification	Experiment	0.856	216.20	0.43	146.18	8.77
		Simulation		217.13		159.01	
	Verification 1	Experiment	0.825	214.46	1.51	144.08	8.95
		Simulation		217.68		156.97	
	Verification 2	Experiment	0.817	233.00	6.17	141.16	11.97
		Simulation		218.61		158.06	
3	Identification	Experiment	0.757	271.43	10.32	159.51	11.11
		Simulation		243.43		177.23	
	Verification 1	Experiment	0.809	292.50	15.85	187.51	4.25
		Simulation		246.14		179.54	
	Verification 2	Experiment	0.789	251.47	2.64	173.68	0.15
		Simulation		244.83		173.95	
4	Identification	Experiment	0.737	101.18	3.20	165.32	5.22
		Simulation		97.94		173.96	
	Verification 1	Experiment	0.759	90.44	7.91	180.16	4.43
		Simulation		97.59		172.17	
	Verification 2	Experiment	0.793	119.79	17.06	167.33	0.75
		Simulation		99.35		168.59	
5	Identification	Experiment	0.834	278.64	11.49	218.57	14.82
		Simulation		310.65		250.95	
	Verification 1	Experiment	0.814	330.93	5.68	204.10	20.19
		Simulation		312.14		245.31	
	Verification 2	Experiment	0.830	277.57	10.33	162.00	14.74
		Simulation		306.26		185.87	

### Influence of muscle synergy on strength identification

**Fig. 6.**
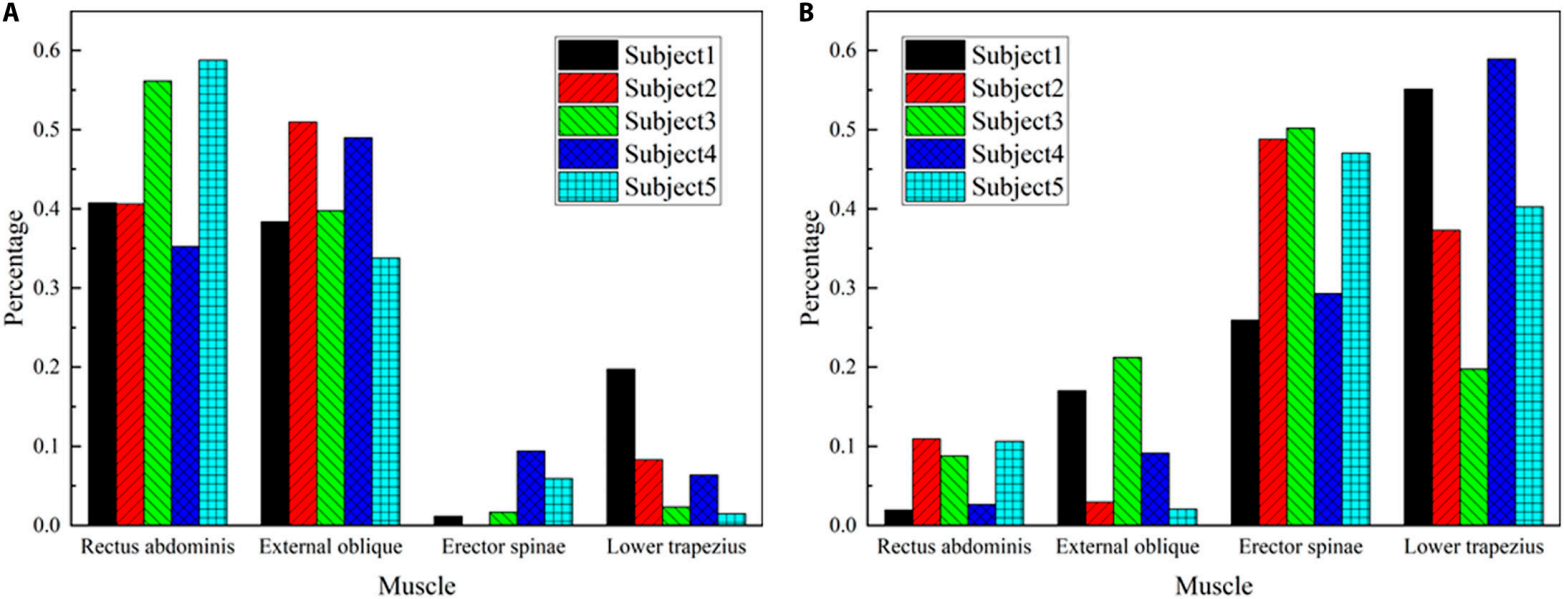
Synergy vectors of spinal muscles extracted based on NMF. (A) Flexor-dominated synergy. (B) Extensor-dominated synergy.

The first two-order synergistic patterns for each subject are shown in Fig. 6. The ES and lower trapezius were less activated within spinal flexor-dominated synergy pattern, and the rectus abdominis and the EOs were less activated within spinal extensor-dominated synergy. As shown in Table 2, the estimated time-dependent spinal moment considering the synergy patterns was more correlated than that without synergy. Using the identified parameters in the first trial as the simulation inputs for the other trials without alternation, the maximum moment errors were less than ±26% of the measured data.

**Table 2.** Comparison of the subject-specific muscle strength identification results considering muscle synergy

Subject	Test		Correlation coefficient	Erector spinae		Rectus abdominis	
				Maximum moment (Nm)	Error (%)	Maximum moment (Nm)	Error (%)
1	Identification	Experiment	0.878	194.30	4.36	170.56	8.00
		Simulation		185.83		156.91	
	Verification 1	Experiment	0.856	181.57	10.14	155.94	12.74
		Simulation		199.99		175.81	
	Verification 2	Experiment	0.890	196.97	25.59	157.02	10.16
		Simulation		146.57		172.97	
2	Identification	Experiment	0.922	216.20	12.00	146.18	3.40
		Simulation		190.25		151.15	
	Verification 1	Experiment	0.902	214.46	10.50	144.08	7.18
		Simulation		191.94		154.42	
	Verification 2	Experiment	0.901	233.00	20.50	141.16	9.44
		Simulation		185.23		154.49	
3	Identification	Experiment	0.850	271.43	13.92	159.51	3.05
		Simulation		233.64		154.65	
	Verification 1	Experiment	0.885	292.50	24.96	187.51	1.25
		Simulation		219.48		185.17	
	Verification 2	Experiment	0.864	251.47	22.56	173.68	0.01
		Simulation		194.74		173.67	
4	Identification	Experiment	0.852	101.18	2.19	165.32	10.96
		Simulation		98.96		147.21	
	Verification 1	Experiment	0.866	90.44	8.75	180.16	19.62
		Simulation		98.35		144.81	
	Verification 2	Experiment	0.923	119.79	10.23	167.33	5.06
		Simulation		132.03		158.87	
5	Identification	Experiment	0.881	278.64	7.23	218.57	12.56
		Simulation		258.48		191.12	
	Verification 1	Experiment	0.876	330.93	19.99	204.10	7.74
		Simulation		264.79		188.31	
	Verification 2	Experiment	0.878	277.57	7.70	162.00	20.04
		Simulation		256.20		194.46	

### Influence of IAP on strength identification

**Fig. 7.**
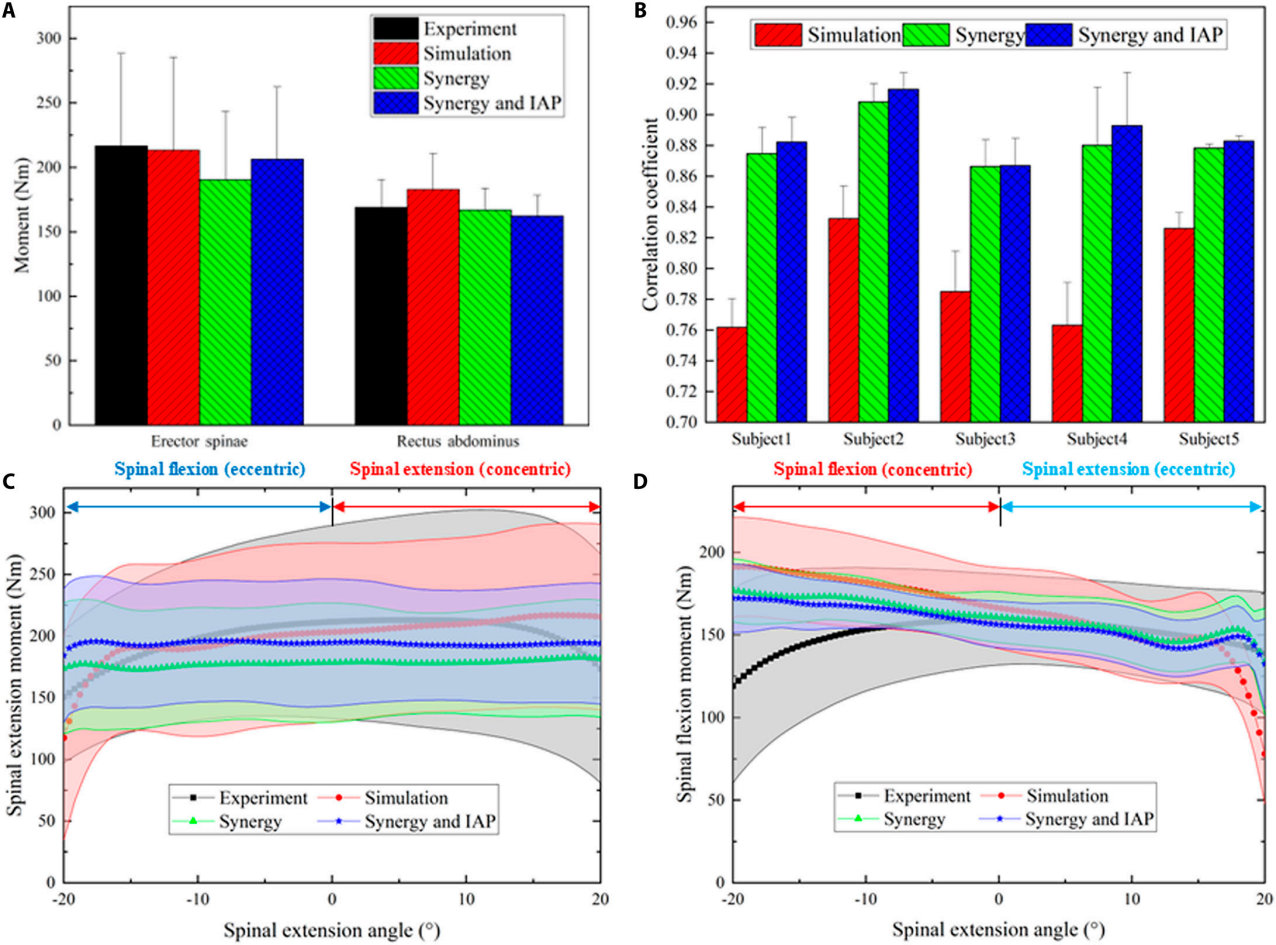
Influence of muscle synergy and IAP on the muscle strength identification. (A) Averaged spinal extensor and flexor moments. (B) Averaged correlation coefficient between the simulated and measured spinal moments. (C) Isokinetic extension moment. (D) Isokinetic flexion moment.

Introducing IAP can increase the identified spinal extensor strength (190.40 ± 53.15 Nm versus 206.30 ± 55.96 Nm) and reduce the identified spinal flexor strength (167.01 ± 16.95 Nm versus 162.29 ± 16.29 Nm) (Fig. 7A). However, abdominal breathing had a tiny effect on the correlation between the simulated and measured spinal moment (Fig. 7B). Moreover, the measured spinal moment reached its maximum value at neutral stance, whereas the simulated curve reached the maximum flexion moment at 20^°^ of spinal flexion and the maximum extension moment at 20^°^ of spinal extension (see Fig. 7C and D).

## Discussion

In this study, the spinal muscle strength identification was performed based on a generic musculoskeletal multibody model rather than a subject-specific one. A subject-specific musculoskeletal model can obtain reliable estimations of muscle strength [53]. However, a detailed subject-specific musculoskeletal model should identify its anatomical and biomechanical parameters from medical imaging, which is difficult for athletes or outpatients. Moreover, the diversity caused by subject-specific modeling could deviate the results of muscle strength identification [54–55].

The PD controller based on the spinal postural parameters was used to solve each muscle’s activation instead of using the optimization solver, which has been commonly used in musculoskeletal dynamics [49,56–57]. It was difficult for static or dynamic optimization methods to design the objective functions for the maximal activation cases like isokinetic testing, and researchers tended to predetermine their muscle activation patterns in isokinetic testing simulations without a control loop [58]. We further assumed that the activation patterns of different fascicles within the same muscle group were identical, reducing the independent control variables. As a consequence, it was impossible to distinguish the activation among different fascicles within the same muscle group, even if they have varied muscle parameters [59].

As the state-of-art approach, muscle activations in musculoskeletal model have been generally derived from the EMG signals. However, the obtained EMG signals might be biased [60] due to the influence of signal crosstalk among multiple muscles [61]. Alternatively, the muscle activation obtained by synergy extraction can better predict muscle force [62]. Previous studies often assumed that the antagonist muscles were not activated during isokinetic loading conditions, and the influence of muscle synergy was rarely considered [63]. The correlation between the simulated and measured moment data could be increased by introducing muscle synergy. More importantly, introducing antagonist muscle activations could improve the joint movement stability [64–65]. Numerous results have shown that the muscle synergy patterns were different between the patients and healthy populations [66–67], and the obtained **W** were sensitive to different movement tasks [68].

Introducing IAP within the musculoskeletal model slightly improved the correlation between simulated and measured moments. However, it did not significantly change the identified spinal extensor strength and cannot reverse the decrease trend caused by including antagonistic synergistic activations. This finding was against the IAP unloading effect reported by other cases [31–32]. According to this study, abdominal breathing mainly improved the spinal stability during isokinetic loading conditions rather than affecting the spinal moment. However, the changes of IAP during isokinetic loading were not monitored and measured in vivo. Further investigations will introduce IAP measurement during isokinetic loading conditions to verify its role in improving spinal stability. Moreover, the optimal muscle fiber lengths of the ES and RA were also adjusted in this study. However, unlike the results reported by Redl et al. [69] and Arslan and Karabulut [70], the alternations of optimal fiber strengths did not strongly influence the identified muscle strength. In other words, in spinal isokinetic conditions, the performed joint moment was strongly influenced by the maximum isometric force, whereas the optimal fiber length had a relatively small effect.

The spinal moment data obtained from the isokinetic measurement and musculoskeletal simulation show a highly diversified individual response. For example, subject 5 had the highest maximum moment in both the spinal flexors and extensors compared with other subjects. Subject 5 was the tallest one with the highest moment arms. Additionally, the subject was the youngest, indicating a lower probability of muscular dystrophy. Subject 4 had lower maximum moments in the ES than other subjects. The subject does not seem to exert maximal moments during the isokinetic testing. Moreover, the identified muscle strength of each subject was higher than the generic model from the OpenSim database. It was possibly because the muscle biomechanical parameters of the generic model were derived from cadaver testing, resulting in a smaller portion of the muscle volume compared with in vivo measurements. Meanwhile, the maximum muscle stress was pre-defined without biomechanical identification [41]. Medical imaging tools, including magnetic resonance imaging or musculoskeletal sonoelastomyography, will be used to personalize musculoskeletal models by identifying muscle fiber length and cross-sectional area.

The limitations of this study are listed. First, the number of recruited subjects was small, and the gender difference was not discussed. The selected electrodes used in this study cannot represent the activation of each muscle-tendon unit within the ES. Further studies could use high-density EMG sensors to identify the difference among each muscle fiber bundle. Meanwhile, the deep muscle activities like the interspinales were assumed to be synergistic with its corresponding superficial ones with higher muscle moment arm. Next, only the spinal extensors and flexors were modeled in our musculoskeletal model [59]. Although the activation level of lower trapezius was measured, its forces were not taken into account within the forward dynamic simulations because the thoracic spine of each subject was fixed by the dynamometer without DOFs. Other muscles important for spinal stabilities, including trapezius and latissimus dorsi [71], were neglected for simplicity. Moreover, our model used bushings to build linear IVD model, but research has shown that IVD exhibits a highly nonlinear response [72–73]. Future research will consider using nonlinear IVD models, whose stiffness and viscosity parameters were obtained from a detailed finite element model like [74].

This study proposed a spinal muscle strength identification framework based on isokinetic testing and flexible musculoskeletal modeling. Simulation results proved that this method can identify the subject-specific spinal extensor and flexor strength with clinically acceptable accuracy. Muscle synergy can improve the correlation between simulated and measured moments, while consideration of abdominal breathing had limited changes in the identified spinal extensor strength. The muscle strength parameters identified in vivo are higher than those from widely used databases, and the obtained spinal muscle strength seems to be influenced by the individual height and age. The established method can help quantify the muscle strength of weightlifting athletes and sarcopenia patients, highlighting its potential in evaluating sports training or rehabilitation outcomes.

## Data Availability

The data are freely available upon request.
